# Comparative Analysis of Chloroplast Genomes in *Cephaleuros* and Its Related Genus (*Trentepohlia*): Insights into Adaptive Evolution

**DOI:** 10.3390/genes15070839

**Published:** 2024-06-26

**Authors:** Jiao Fang, Lingling Zheng, Guoxiang Liu, Huan Zhu

**Affiliations:** 1 Wuhan Institute of Biomedical Sciences, School of Medicine, Jianghan University, Wuhan 430056, China; fangjiao@jhun.edu.cn; 2 Key Laboratory of Algal Biology, Institute of Hydrobiology, Chinese Academy of Sciences, Wuhan 430072, China; zhengll@ihb.ac.cn (L.Z.); liugx@ihb.ac.cn (G.L.)

**Keywords:** organelle genome, *Cephaleuros*, chloroplast, comparative analysis, positive selection

## Abstract

*Cephaleuros* species are well-known as plant pathogens that cause red rust or algae spot diseases in many economically cultivated plants that grow in shady and humid environments. Despite their prevalence, the adaptive evolution of these pathogens remains poorly understood. We sequenced and characterized three *Cephaleuros* (*Cephaleuros lagerheimii*, *Cephaleuros diffusus*, and *Cephaleuros virescens*) chloroplast genomes, and compared them with seven previously reported chloroplast genomes. The chloroplast sequences of *C. lagerheimii*, *C. diffusus*, and *C. virescens* were 480,613 bp, 383,846 bp, and 472,444 bp in length, respectively. These chloroplast genomes encoded 94 genes, including 27 tRNA genes, 3 rRNA genes, and 64 protein-coding genes. Comparative analysis uncovered that the variation in genome size was principally due to the length of intergenic spacer sequences, followed by introns. Furthermore, several highly variable regions (*trnY-GTA*, *trnL-TAG*, *petA*, *psbT*, *trnD-GTC*, *trnL-TAA*, *ccsA*, *petG*, *psaA*, *psaB*, *rps11*, *rps2*, and *rps14*) were identified. Codon bias analysis revealed that the codon usage pattern of *Cephaleuros* is predominantly shaped by natural selection. Additionally, six chloroplast protein-coding genes (*atpF*, *chlN*, *psaA*, *psaB*, *psbA*, and *rbcL*) were determined to be under positive selection, suggesting they may play a vital roles in the adaptation of *Cephaleuros* to low-light intensity habitats.

## 1. Introduction

The genus *Cephaleuros* Kunze, from the order Trentepohliales, consists of approximately 17 species found in tropical and subtropical regions [1,2,3]. *Cephaleuros* species are well-known plant pathogens, parasitic or endophytic in many economically important plants, such as *Lansium parasiticum*, *Psidium guajava*, *Dimlongan*, blackberry, and tea trees [4,5,6,7,8]. Plants infected by *Cephaleuros* species reduce the photosynthetic area of the leaves and damage their aesthetic value [1]. Moreover, lesions on the surface of fruits, such as guava, may affect their marketability [5]. Members of *Trentepohlia* are free-living, with several species identified as phycobionts in lichens [9]. The distinct habitat characteristics of two genera are not only important taxonomic traits that distinguish them, but also make them excellent taxa for the study of adaptive evolution.

Plastids are organelles that are unique to plant and algae. They are essential for plant growth and development, including carbon fixation, ATP synthesis, photosynthesis, and lipid biosynthesis [10]. The plastid genome has developed into an essential molecular tool for studying plant adaptive evolution. For example, three plastid genes (*rpl16*, *ndhA*, and *ndhH*) under positive selection may be involved in the adaptation of *Rhodiola* to low CO_2_ levels and high-intensity light environments [11]. Similarly, positive selection on nine plastid genes may help *Paraboea* species adapt to harsh karst environments [12]. Recently, the positive selection of the *atpA* and *ycf2* genes may help *Meconopsis* adapt to the low CO2 concentration and cold conditions of the plateau habitats [13]. Environmental stress may apply selective pressure to genes, leaving a natural selection footprint on chloroplast genomes. However, the adaptive evolution of *Cephaleuros* species is still poorly known.

We successfully sequenced three *Cephaleuros* chloroplast genomes. We then compared these chloroplast genomes with previously published genomes to investigate genome traits and adaptive evolution in the *Cephaleuros* and *Trentepohlia* genera. Furthermore, the hotspot regions in the chloroplast genomes were identified. Selective pressure analyses were conducted to determine whether certain genes were subject to purifying or positive selection in *Cephaleuros*. Finally, phylogenetic relationships were constructed using chloroplast protein-coding sequences and ITS rDNA to verify the genetic relationship of the sequenced chloroplast genomes in genera *Cephaleuros* and *Trentepohlia*. Our results provide insights into the adaptive evolution of *Cephaleuros*.

## 2. Materials and Methods

### 2.1. Sample Collection and DNA Extraction

*C. lagerheimii* was collected from Qingxiu Mountain in Nanning City, Guangxi Province. *C. diffusus* was obtained from the Wuhan Botanical Garden, Chinese Academy of Sciences in Wuhan City, Hubei Province, and *C. virescens* from Longkong Cave in Longyan City, Fujian Province. Voucher specimens were identified by Jiao Fang and Huan Zhu based on previous articles [2,3,9]. These specimens were stored at the herbarium of the Institute of Hydrobiology (Wuhan, China), Chinese Academy of Sciences (FACHB) under deposition numbers FACHB-3599, FACHB-3600, and FACHB-3601, respectively. Three samples were cultivated in BBM solid culture dishes containing 1.2% agar. The culture conditions were 12 h light and 12 h dark cycle, and the illumination intensity was 35–50 μmol photons m^−2^ s^−1^. A constant 20 °C temperature was maintained within a dedicated culture chamber.

The algal thalli were initially merged with 1 mL ceramic beads (0.5 mm) and 350 μL of phosphate buffer solution (pH 7.0), followed by lysis using a Bioprep-24 Homogenizer (BH-24; Hangzhou Miu Instruments Co., Ltd., Hangzhou, China) at 4000 rpm for 2 min. The UE DNA preparation kit (US Everbright, Suzhou, China) was used to obtain total DNA. The primers and PCR amplification settings for the ITS rDNA were described in a previous article [2]. The results of PCR were subsequently delivered to Sangon Biotech in Shanghai, China, for sequencing. The ITS sequences of *Cephaleuros diffusus* were deposited in GenBank with accession number PP033476. The ITS sequences of *C. lagerheimii* and *Cephaleuros virescens* were retrieved from the National Center for Biotechnology Information (NCBI) website (https://www.ncbi.nlm.nih.gov/, accessed on 20 November 2022).

### 2.2. Chloroplast Genome Assembly and Annotation

The genomic DNA was acquired using the CTAB technique [14]. Subsequently, sequencing was executed using an Illumina NovaSeq 6000 at the Benagen company in Wuhan, China. To ensure the reliability of the data, it was essential to filter the raw data for low-quality sequences and remove adapters. We utilized the SOAPnuke 2.X version to filter the raw data, resulting in the high-quality and clean reads [15]. This critical step was taken to ensure the reliability and accuracy of subsequent analyses. SPAdes 3.15.5 was then used to assemble the clean reads [16].

The annotation of chloroplast genomes was executed utilizing GeSeq (https://chlorobox.mpimp-golm.mpg.de/geseq.html, accessed on 18 December 2022) [17]. Additionally, the refinement of protein-coding and rRNA genes was achieved through BLAST alignment with the chloroplast sequence of *Trentepohlia odorata*. The identification of tRNA genes was validated by tRNAScan-SE [18]. Chloroplast genomic maps were generated with OGDRAW [19]. The annotated chloroplast sequences of *C. virescens*, *C. lagerheimii*, and *C. diffusus* were subsequently uploaded to the NCBI, with numbers OQ848499, OQ848500, and OQ848501, respectively (Table 1).

### 2.3. Genome Comparison and Divergent Hotspots Identification

Seven sequences from the *Cephaleuros* and *Trentepohlia* genera were obtained from the NCBI, including three sequences from *Cephaleuros* and four from *Trentepohlia* (Table 1). Whole genome alignments were performed using progressiveMauve, which was integrated into the Mauve version 20150226, employing the default settings [20]. Double-cut-and-join (DCJ) genome distances were calculated using UniMoG [21]. Furthermore, to identify variable regions within the two genera, nucleotide diversity (Pi) values were obtained for common genes using DnaSP v6 [22].

### 2.4. Codon Bias Analyses

Parameters related to codon bias analysis were obtained employing CodonW 1.4.2 (https://codonw.sourceforge.net/culong.html#CodonW, accessed on 25 December 2022). These included T3, C3, A3, and G3, representing the frequencies of T, C, A, and G usage at the third base of the codons. Additionally, GC3s refers to the GC content in the third position of the synonymous codons, and the ENc (effective number of codons) was also calculated using CodonW 1.4.2.

The GC content of the first (GC1), second (GC2), and third codon (GC3) positions was determined using the online CUSP tool (https://www.bioinformatics.nl/emboss-explorer/, accessed on 25 December 2022).

The parity rule 2 (PR2) plot was utilized to explore the nucleotide composition at the third position of codons. This commonly adopted method facilitates the determination of whether mutation pressure or selection pressure dominates the nucleotide composition in DNA double strands. In the PR2 plot, the horizontal and vertical coordinates represent G3/(G3+C3) and A3/(A3+T3), respectively. If only mutation pressure influences the codons, the result is all the points being in the center of the plot. Alternatively, if codon usage is influenced by natural selection and other factors, it causes the points to deviate from the center of the plot [23].

In the ENc-GC3s plot, the GC3s is plotted on the horizontal axis, while the actual ENc values (actENc) are plotted on the vertical axis. Notably, codons encoding methionine (Met) and tryptophan (Trp) were excluded due to their absence of synonymous codons. The expected ENc (expENc) values were obtained using the following formula:

$$\mathrm{expEN}c=2+\mathrm{GC}3s+\frac{29}{{\mathrm{GC}3s}^{2}+{\left(1-\mathrm{GC}3s\right)}^{2}}$$

A standard curve was generated based on the expected values. Data points that lie on the standard curve indicate a significant influence of mutational pressure on codon usage. Conversely, data points significantly below the standard curve indicate that natural selection and other factors may be the primary driving forces influencing codon usage patterns [24]. ENC ratio (ENcratio) was determined using the following formula, which illustrates the difference between the actENc and expENc values:

$$\mathrm{ENcratio}=\frac{(\mathrm{expENc}-\mathrm{actENc})}{\mathrm{expENc}}$$

Neutrality plot analysis was conducted to investigate the extent of influence between mutation pressure and natural selection on codon usage patterns. GC12 (the average GC content at the first and second positions of codon), GC1, GC2, and GC3 were analyzed. The GC3 content was plotted on the horizontal axis, while the GC12 content was plotted on the vertical axis, resulting in two-dimensional scatter plots representing individual genes. In the neutrality plot, if the slope of the regression curve approaches 0, and there is no significant correlation between GC12 and GC3, it shows that natural selection is the primary factor driving codon usage patterns. Conversely, when the slope is close to or equal to 1, and a significant correlation exists, it indicates that mutation pressure is likely to exert significant influence on gene evolution [25].

### 2.5. Selective Pressure Analyses

A selective pressure analysis was performed on 10 chloroplast genomes of Trentepohliales, employing three distinct calculations. Firstly, pairwise Ka/Ks ratios were calculated based on concatenated protein-coding genes. Secondly, Ka/Ks ratios were determined as the single protein-coding gene. Finally, the branch-site model was employed for further analysis. Sixty-four protein-coding genes were obtained and concatenated into a super array using PhyloSuite v1.2.3 [26]. Each gene was aligned by muscle (codons) pattern in MEGA 6 [27]. The KaKs_Calculator v2.0 was applied to determine the pairwise Ka/Ks ratios for each species [28]. Similarly, Ka/Ks ratios were calculated for each protein-coding gene independently.

The ratio between nonsynonymous and synonymous substitution rates (ω) was employed to assess the selective pressure. This ratio provides insights into the presence of negative purifying selection (0 < ω < 1), neutral evolution (ω = 1), or positive selection (ω > 1). To identify potential positive selection, we employed the branch-site model within the CODEML tool of the PAML v4.9 [29]. In this analysis, we focused on 54 single-copy genes extracted from chloroplast genomes, with the genus *Cephaleuros* designated as the foreground branch.

Two models were employed within the branch-site analysis: a null branch-site model (model = 2, NSsites = 2, fix_omega = 1, omega = 1) and an alternative branch-site model (model = 2, NSsites = 2, fix_omega = 0, omega = 2). A statistical likelihood ratio test (LRT) was employed to evaluate the potential for positive selection by comparing the alternative branch-site model to the null branch-site model. The *p*-values were calculated using the Chi-square program in PAMLX v1.3.1 [30]. Furthermore, a Bayes Empirical Bayes (BEB) analysis was performed to assess the posterior probabilities of positive selection sites [31]. Sites with BEB values > 0.95 and *p*-values < 0.05 were considered to be positively selected. Finally, the PSIPRED server was employed to visualize the amino acid sequences and their corresponding secondary structures in positively selected genes [32]. Additionally, the protein structures of these genes were predicted using SWISS-MODEL online web-server [33].

### 2.6. Phylogenetic Inference

Phylogenetic inferences were constructed, based on the ITS rDNA and chloroplast protein-coding sequences, to verify the identity of the strains. In the first phylogenetic analysis, *Trentepohlia prolifera* KX586859 and *Trentepohlia* sp. KX586863, downloaded from GenBank, were selected as the outgroup. Alignment of all sequences was achieved using MAFFT v7.520 [34], and unclear regions were subsequently trimmed through TrimAl v1.2 with the -gt 0.8 option [35]. The best models were detected using ModelFinder [36].

A phylogenetic tree was constructed for the core Chlorophyta, comprising 102 core Chlorophyta species, with 5 Prasinophyte species selected as outgroups. In order to facilitate phylogenetic reconstruction, 31 conserved protein-coding genes identified in a previous study were used [37], encompassing *atpA*, *atpB*, *atpF*, *atpH*, *rps7*, *rps8*, *rps9*, *petB*, *petD*, *petG*, *psaB*, *psaC*, *clpP*, *rbcL*, *rpl2*, *rpl5*, *rpl14*, *rpl16*, *rpl20*, *rps11*, *rps18*, *rps19*, *tufA*, *ycf3*, *psbA*, *psbB*, *psbJ*, *psbK*, *psbM*, *psbN*, and *psbZ*.

Protein-coding genes were extracted by PhyloSuite v1.2.3 [26]. Sequences were aligned utilizing MAFFT v7 with the ‘-auto’ strategy and codon alignment mode [34]. The alignments were optimized using MACSE v2.06 to obtain a final alignment [38]. The Gblocks program was employed to eliminate the ambiguously aligned regions of alignments [39]. Subsequently, the Concatenate Sequence tool in PhyloSuite v1.2.3 was used to concatenate the aligned sequences [26].

Phylogenetic analyses were conducted employing Bayesian inference (BI) and maximum likelihood (ML) methods. The software MrBayes v3.2.6 and the online tool IQ-TREE (https://www.hiv.lanl.gov/content/sequence/IQTREE/iqtree.html, accessed on 27 December 2022) were employed for the BI and ML analyses, respectively [40,41]. ModelFinder was employed to identify the optimal partition model based on the Bayesian Information Criterion (BIC) [36]. The ML analysis was performed using 1000 bootstrap replicates. The BI tree was performed for 8,000,000 generations in the ITS rDNA. The first quarter of the trees were burned in. The remaining trees were harnessed for the generation of a consensus tree. The resulting phylogenetic trees were visualized using FigTree v1.4.2 (http://tree.bio.ed.ac.uk/software/figtree/, accessed on 27 December 2022).

## 3. Results

### 3.1. Three New Cephaleuros Chloroplast Genomes

**Figure 1:**
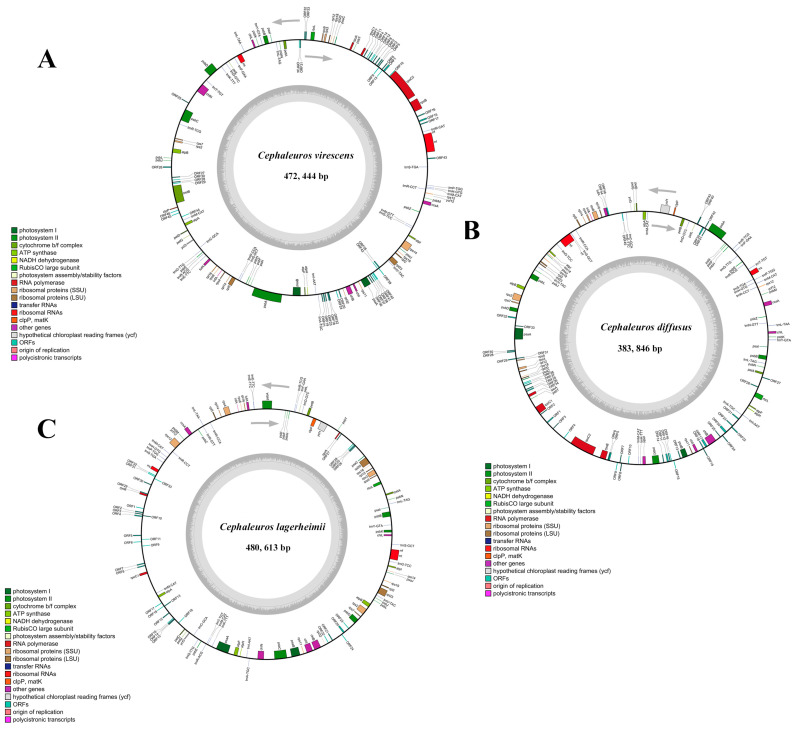
Gene maps of three *Cephaleuros* chloroplast genomes: (**A**) *Cephaleuros virescens*; (**B**) *Cephaleuros diffusus*; and (**C**) *C. lagerheimii*. The clockwise arrow denotes the direction of transcription of genes inside the circle. The counterclockwise arrow denotes the direction of transcription of genes outside the circle. The GC content is shown in dark gray, and the AT content is shown in light gray. Genes are annotated with various colors according to the different functions.

The assembled plastid lengths for *C. lagerheimii* (OQ848500), *C. diffusus* (OQ848501), and *C. virescens* (OQ848499) were 480,613 bp, 383,846 bp, and 472,444 bp, respectively. The 3 sequenced chloroplast genomes exhibited a circular molecule, with no inverted repeat (IR) regions (Figure 1). Within these 3 *Cephaleuros* genomes, 94 genes were determined, including 64 protein-encoding genes, 27 tRNA genes, and 3 rRNA genes (Table 1 and Table S1). The total GC contents of *C. lagerheimii*, *C. diffusus*, and *C. virescens* were 29.5%, 33.4%, and 33.7%, respectively. The *rpoC2* was absent in *C. lagerheimii*, the *ycf1* was missing in *C. virescens*, and the *rpl32* was completely absent in *C. diffusus*.

**Figure 2:**
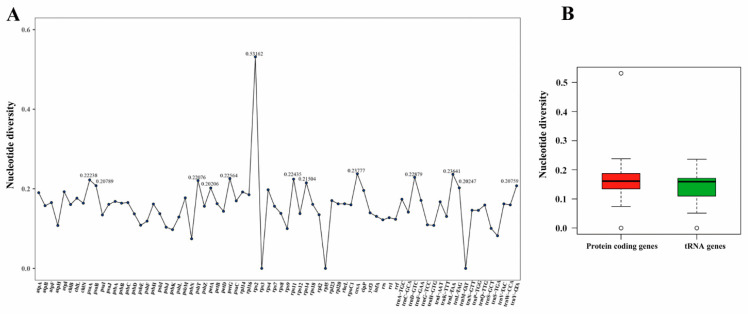
The variation of nucleotide diversity among the genera *Cephaleuros* and *Trentepohlia*: (**A**) nucleotide diversity values (Pi) among the 10 sequenced chloroplast genomes and (**B**) comparison of Pi values between chloroplast coding genes and tRNA genes. The bold solid black line in the graph represents the location of the median.

The results of the nucleotide diversity analysis, based on 77 common genes, are depicted in Figure 2A. The average pairwise sequence divergence (Pi) value was 0.15699, and Pi values varied from 0 to 0.53162. Notably, 13 genes (*trnY-GTA*, *trnL-TAG*, *petA*, *psbT*, *trnD-GTC*, *trnL-TAA*, *ccsA*, *petG*, *psaA*, *psaB*, *rps11*, *rps2*, and *rps14*) exhibited relatively high Pi values (Figure 2A). These highly variable loci may prove useful for phylogenetic inference. In general, protein-coding genes displayed more polymorphisms compared to tRNA genes (Figure 2B).

### 3.2. Comparative Analysis

A comparative analysis based on chloroplast genomes was conducted within the genus *Cephaleuros*. In addition to the newly sequenced genomes, three previously published *Cephaleuros* chloroplast genomes, accessible in the NCBI database, were included in the analysis. The lengths of *Cephaleuros* chloroplast genomes ranged from 266,729 bp (*C. parasiticus*) to 480,613 bp (*C. lagerheimii*), and the three sequenced chloroplast genomes have no inverted repeat regions (Table 1). The number of genes ranged from 94 to 98.

**Figure 3:**
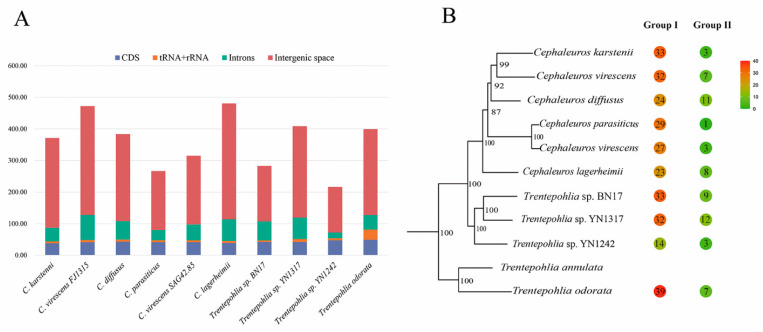
(**A**) Variation of chloroplast genomes size in the genera *Cephaleuros* and *Trentepohlia*. The plastid protein-coding regions are conserved, and differences in genome size are primarily explained by intergenic space and introns. (**B**) Group I introns and group II introns of *Cephaleuros* and *Trentepohlia*. In the colored circles, red represents a higher number of introns, while green represents a lower number of introns. The number inside the circle represents the number of introns.

Furthermore, comparative analysis of the genera *Cephaleuros* and *Trentepohlia* was performed. Only *T. odorata* exhibited a quadripartite structure, comprising the large single-copy (LSC) sequence, the small single-copy (SSC) sequence, and two inverted repeats (IRs). However, in *Trentepohlia* sp. YN1242 and *Trentepohlia* sp. YN1317, as well as in the genus *Cephaleuros*, the IR region had been lost. The sequenced chloroplast genomes of two genera ranged in length from 216,308 bp to 480,613 bp. The variation of chloroplast genome length was primarily attributed to differences in intergenic regions, followed by introns (Figure 3A). The genes in these genomes ranged from 93 to 97, including 3 rRNA genes, 25 to 30 tRNA genes, and 63 to 65 protein-coding genes. The range of the total GC content was 25.9% to 36.1% (Table 1).

Intron variation played a crucial role in chloroplast genome size (Figure 3A). The chloroplast introns were identified in both rRNA and protein-coding genes, but not in tRNA genes. Notably, the number of group I introns was significantly higher than that of group II introns (Figure 3B). Among the species analyzed, only *T. odorata*, which possessed the inverted repeats (IRs), exhibited the highest number of group I introns. Interestingly, the presence of group I introns in the *rpl2* gene was exclusive to *Trentepohlia* algal strains (Figure S1). Furthermore, group I introns were identified in the *psbC* and *rrl* genes of all sequenced chloroplast genomes, and all introns in rRNA were classified as group I.

More than 50 local collinear blocks were found, revealing significant rearrangements and inversions within *Cephaleuros* (Figure S2). Notably, the highly divergent regions were primarily located in the intergenic regions, such as *trnM–rpoB*, *rpoC2–rpoC1*, *chlN–psbC*, *atpB–psbL*, *psbJ–petB*, and *trnM–atpA*. Additionally, some divergences were observed in the coding regions, including *psaC–rpl20–rps18–rpl12–rps3–rps9*, *rps7–rps2*, *psbB*, *psbK*, and *petD*. Genome rearrangements provide insights into evolutionary dynamics at the genomic level. The lowest DCJ value (10) was observed between *C. virescens* SAG 42.85 and *C. parasiticus* GD1927, while the highest value (41) was found among different *C. virescens* algae strains (Table S2). Similarly, synteny analysis of the genera *Cephaleuros* and *Trentepohlia* revealed considerable inversions and rearrangements (Figure S3). The DCJ values for pair-to-pair comparisons among two genera are presented in Table S3. The highest DCJ value (69) was observed between *C. parasiticus* and *T. odorata*, while the DCJ values between *T. odorata* and *C. virescens* SAG 42.85 was 68 (Table S3).

### 3.3. Codon Usage Analyses

**Figure 4:**
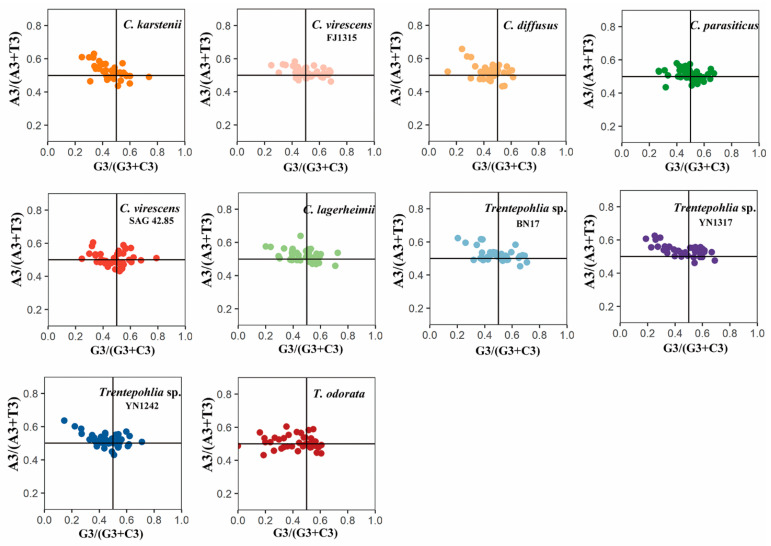
PR2 plot of chloroplast genomes of the genera *Cephaleuros* and *Trentepohlia*. If there is no codon usage bias, A=T and C=G, and the point lies at the center of the graph. The first quadrant represents codon bias towards A/G at the third position of the codon, while the third quadrant represents a preference for T/C at the third position of the codon. The different coloured dots in the figure represent genes from different species.

In PR2 plot analysis, the expected pattern under the influence of mutation pressure alone is that the frequencies of nucleotides A and T are equal to that of C and G at the third codon position. This causes the genes to cluster in the center of the plot. Conversely, if natural selection is at play, the relative usage of A and T bases would differ from that of G and C nucleotides, resulting in genes deviating from the center of the plot. The scatter plots representing the A3/(A3+T3) and G3/(G3+C3) values are depicted in Figure 4. The A/T-bias values were determined as 0.522, 0.524, 0.520, 0.514, 0.517, 0.517, 0.508, 0.523, 0.518, and 0.537 for *C. diffusus*, *C. karstenii*, *C. lagerheimii*, *C. parasiticus*, *C. virescens* (FJ1315), *C. virescens* (SAG 42.85), *T. odorata*, *Trentepohlia* sp. (BN17), *Trentepohlia* sp. (YN1242), and *Trentepohlia* sp. (YN1317), respectively. The G/C-bias values were 0.443, 0.461, 0.474, 0.484, 0.503, 0.483, 0.416, 0.495, 0.455, and 0.463, respectively. Notably, the distribution of coding sequences (CDSs) did not exhibit an even distribution around the center point (A=T, G=C). The majority of genes were positioned above the horizontal centerline of 0.5, indicating a ratio of A3/(A3+T3) > 0.5, particularly observed in *Trentepohlia* species. These findings indicate a bias towards A and C at the third position of codons in chloroplast genes, suggesting a significant influence of selection pressure on codon usage patterns. Nevertheless, it is important to note that a few genes were positioned close to the center, illustrating that the mutation pressure cannot be disregarded.

**Figure 5:**
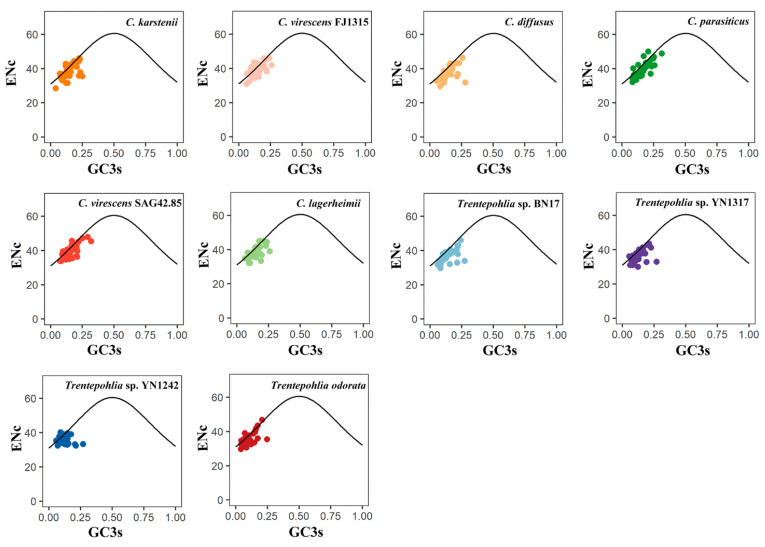
ENc plot of chloroplast genomes of the genera *Cephaleuros* and *Trentepohlia*. ENC denotes the effective number of codons, and GC3s denotes GC content in the third position of synonymous codons. The standard curve represents the expected ENC values. Points on or near the curve suggest bias caused by mutation pressure. Points that deviate from the curve suggest bias influenced by natural selection or other factors. The different coloured dots in the figure represent genes from different species.

**Figure 6:**
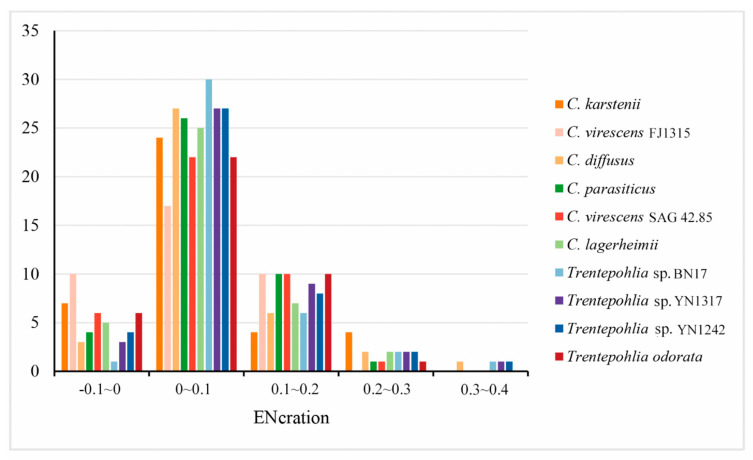
Distribution of ENC frequency of chloroplast genomes in the genera *Cephaleuros* and *Trentepohlia*.

In an ENc plot, the points located on the standard curve indicate an influence of mutation pressure on codon usage. Conversely, the points that deviate from the standard curve suggest that natural selection and other factors may be the primary driving forces influencing codon usage patterns. The distributions of ENc and GC3s in the 10 chloroplast genomes exhibit similarity (Figure 5). Some points are dispersed along or around the standard curve, while others are below the standard curve. These results indicate that the codon usage bias of the chloroplast genomes of the two genera is influenced not only by mutation pressure but also by natural selection and other factors. The ENC frequency of chloroplast genes in the 10 sequenced chloroplast genomes was calculated to better observe the difference between the actENC and expENC value (Figure 6). In Figure 6, most of the ENC ratios were distributed between 0~0.1, indicating that the actual ENC were slightly smaller than expected. The results verified that most of the points in Figure 5 were below the standard curve, suggesting that mutation pressure might be a weak factor affecting the evolutionary history of the two genera.

**Figure 7:**
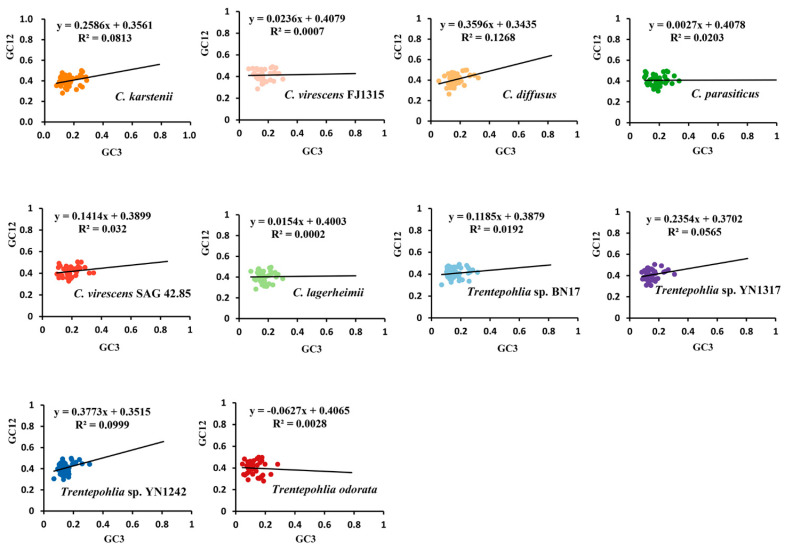
Neutrality–plot of chloroplast genomes of the genera *Cephaleuros* and *Trentepohlia*. GC12 represents the average GC content at the first and second positions of the codons. GC3 represents the GC content in the third position of codons. The black solid line represents the regression line. The equation of the regression line is shown at the top of each plot. The different coloured dots in the figure represent genes from different species.

In the neutrality plots, these genes had a narrow range of GC12 (0.26~0.51) and GC3 (0.04~0.345) values (Figure 7). There was no significant correlation between GC12 and GC3 in the 10 sequenced chloroplast genomes (rck = 0.2851, rcv = 0.0265, rcd = 0.3561, rcp = 0.1425, rcv4285 = 0.1789, rcl = 0.0141, rT17 = 0.1386, rT1317 = 0.2377, rT1242 = 0.3161, and rTo = 0.0529), which indicated that mutation pressure had a slight effect on the codon usage bias. Among the 10 chloroplast genomes, the slopes of the regression lines were 0.2586, 0.0236, 0.3596, 0.0027, 0.1414, 0.0154, 0.1185, 0.2354, 0.3773, and −0.0627. This indicates that the mutation pressure effect accounted for only 0.27% to 37.73%, highlighting the significant role of natural selection. The results demonstrate that codon usage bias is slightly affected by mutation pressure, while natural selection and other factors may play important roles.

### 3.4. Selective Pressure Analyses

**Figure 8:**
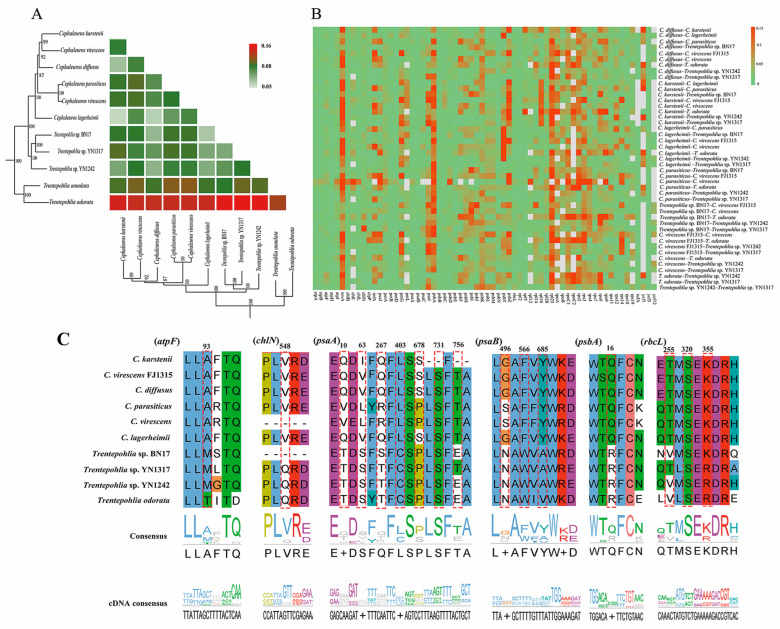
(**A**) Pairwise Ka/Ks ratios among the *Cephaleuros* and *Trentepohlia* species. Heatmap denotes pairwise Ka/Ks ratios between every sequence in the multigene nucleotide alignment. (**B**) Pairwise Ka/Ks ratios of different genes of *Cephaleuros* and *Trentepohlia* species. Heatmap indicates pairwise Ka/Ks ratios among each individual gene in the 10 sequenced chloroplast genomes. Grey represents missing genes so that the Ka/Ks ratio cannot be calculated. (**C**) The amino acids sequences of six genes of positive selection. The red dashed lines denote the amino acids with a high BEB posterior probability in *Cephaleuros* and *Trentepohlia* species.

We conducted Ka/Ks calculations to assess selective pressures within the 10 sequenced chloroplast genomes by constructing a super-matrix from all 64 coding sequences. The majority of Ka/Ks values fell within the range of 0.05 to 0.2, indicating evidence of purifying selection on these chloroplast genes (Figure 8A, Table S4).

Additionally, we calculated Ka/Ks values individually for all 64 protein-coding genes (Figure 8B, Supplementary Table S5). Among these genes, three (*psbA*, *psbT*, and *rps9*) exhibited Ka/Ks values around 0.5 in certain species, suggesting the possibility of positive selection. Most of the other genes exhibited Ka/Ks values ranging from 0.01 to 0.1, indicating strong purifying selection (Supplementary Table S5).

To further determine the chloroplast protein-coding genes that potentially underwent positive selection in the 10 sequenced chloroplast genomes, a branch-site model analysis was conducted for 54 single-copy genes, and the free-living *Trentepohlia* species were selected as foreground branches. The result indicated six protein-coding genes (*atpF*, *chlN*, *psaA*, *psaB*, *psbA*, and *rbcL*) have been under positive selection, most of which are related to photosynthesis (e.g., *psaA*, *psaB*, *psbA*) (Figure 8C, Table S6).

**Figure 9:**
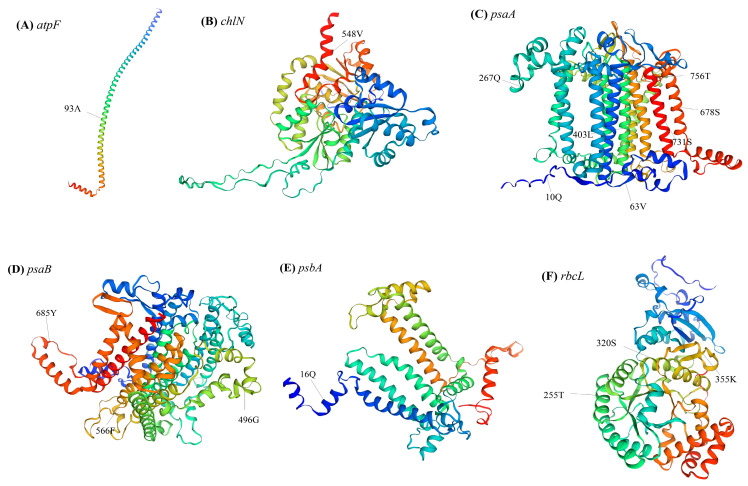
Spatial distribution of the positively selected sites: (**A**) spatial distribution of the positively selected sites in the *atpF*; (**B**) spatial distribution of the positively selected sites in the *chlN*; (**C**) spatial distribution of the positively selected sites in the *psaA*; (**D**) spatial distribution of the positively selected sites in the *psaB*; (**E**) spatial distribution of the positively selected sites in the *psbA*; and (**F**) spatial distribution of the positively selected sites in the *rbcL*.

Bayes Empirical Bayes analysis identified 16 sites with significant posterior probabilities, indicating positive selection. Among the identified sites, six exhibited Bayes Empirical Bayes posterior probabilities exceeding 0.99, the remaining sites exhibited probabilities exceeding 0.95. Three genes (*atpF*, *chlN*, and *psbA*) had only one positively selected site, whereas *psaA*, *psaB*, and *rbcL* contained 7, 3, and 3 positively selected sites, respectively. Notably, in the positively selected sites of *atpF*, all *Cephaleuros* species encoded alanine, *Trentepohlia* species without IR encoded methionine, and species containing IR encoded threonine (Figure 8C). This further elucidates the specificality of *Cephaleuros*. In the *psaA* gene, all *Trentepohlia* chloroplast genomes encoded the same amino acids at the 10th and 63rd sites, while the amino acids encoded in *Cephaleuros* were different. At the 403rd site, all *Cephaleuros* encoded leucine (L), while all *Trentepohlia* encoded cysteine (C). A similar pattern was observed at the 566th and 685th sites of *psaB* and the 16th site of *psbA*, where all *Trentepohlia* encoded the same amino acid, whereas all *Cephaleuros* encoded another identical amino acid. All 10 sequenced species encoded serine at the 731st site of *psaA* and the 320th site of *rbcL*, but variations occurred at the codon level (Figure 8C). Furthermore, the spatial analysis of the *atpF* protein revealed that the site under positive selection was inside in the α-helix (Figure 9A). The positive selection sites of both the *chlN* and *psbA* genes were also located in the α-helix. Four amino acid sites (403rd, 678th, 731st and 756th) under positive selection in *psaA* were found in the α-helix, two amino acid sites (10th and 63rd) were located in a random coil, and one amino acid site (267th) was located in β-turn. Three amino acid sites (496th, 566th and 685th) that were subject to positive selection in *psaB* were located in a random coil. One amino acid site (255th) under positive selection in *rbcL* was situated in an α-helix and two sites were displayed in a random coil.

### 3.5. Phylogenetic Inferences

The first phylogenetic tree based on ITS rDNA was constructed to confirm the identity of our algal strains. The best-fit models for the Bayesian inference (BI) and maximum likelihood (ML) analyses were K2P+G4 and GTR+F+G4, respectively. The phylogenetic trees constructed by the ML and Bayesian methods showed similar topologies. The phylogenetic analysis supported that *C. diffusus* HB1902 was located within the *C. diffusus* branch with a strong support value (Figure S4). *C. virescens* FJ1315 was found to cluster with other *C. virescens* downloaded from the NCBI database. *C. lagerheimii* was distinguished from other *Cephaleuros* species and formed a single branch (Figure S4).

The phylogenetic relationships of the three *Cephaleuros* species were inferred, based on 31 chloroplast protein-coding genes, using Prasinophytes as an outgroup. The best-fit models for the maximum likelihood (ML) analysis are presented in Table S7. *C. virescens* FJ 1315 was found to cluster with the morphologically similar *C. karstenii*, and *C. diffusus* was sister to *C. virescens* and *C. karstenii* (Figure S5). Furthermore, this result showed that *Cephaleuros virescens* was not monophyletic. *C. lagerheimii* formed a distinct branch, which was consistent with the phylogeny of ITS rDNA (Figures S4 and S5).

## 4. Discussion

### 4.1. Variations in Chloroplast Genomes

*Cephaleuros* species are parasitic, and *Trentepohlia* species are free-living. Their distinct habitats make them excellent materials for studying adaptive evolution. Previous studies have reported three *Cephaleuros* chloroplast genomes and four *Trebtepohlia* chloroplast genomes, and compared them with other Ulvophyceae taxa [37,42]. However, few studies have been conducted on codon usage bias and adaptive evolution of these two genera with distinct habitats. We present the chloroplast genomes of *C*. *lagerheimii* and *Cephaleuros diffusus* for the first time and add the chloroplast genome data of *C. virescens*, which facilitate the analysis of *Cephaleuros* adaptive evolution. We then compared these chloroplast genomes with previous published genomes [37,42], and conducted adaptive evolution analyses in *Cephaleuros* and *Trentepohlia*.

Chloroplast genomes typically exhibit highly conserved structures, consisting of a circular molecule containing a large single-copy (LSC) region, a small single-copy (SSC) region, and a pair of inverted repeats (IRs) [43]. In this study, three sequenced genomes had no IR region, which was consistent with previously released *Cephaleuros* chloroplast genomes [37]. The IR regions of some organisms were absent, which seems to be common in green algae groups, such as *Cephaleuros* (this article), Bryopsidales, Chaetophorales, and Watanabeales [44,45,46]. The chloroplast genomes of the parasitic habitat-dwelling *Cuscuta* also lacked inverted repeat regions [47]. Moreover, the plastid genome of *Chromera velia* was linear [48], the Cladophorales chloroplast genome was entirely broken into hairpin chromosomes [49], and the chloroplast genome of dinoflagellates exhibited an unusual splitting into minicircles ranging from 2 to 10 kb in size [50].

Photosynthetic land plant plastid genomes range from 120 to 160 kb [51]. The chloroplast genomes of *Cephaleuros* and *Trentepohlia* species exhibit a broader size range, spanning from 216,308 to 480,613 bp. Notably, 9 out of the 10 sequenced chloroplast genomes exceeded 250 kb in size (Table 1). The significant differences in genome length are mainly due to variations in the length of intergenic regions (Figure 3A). For instance, the largest chloroplast genome is more than two times larger than the smallest chloroplast genome in the two genera. This is primarily due to discrepancy in the length of intergenic regions. The chloroplast genome of *Floydiella terrestris*, which is found in soil habitats, was the largest chloroplast genome ever sequenced (521,168 bp). Intergenic regions accounted for 77.8% of its genome length [52]. Furthermore, the intergenic regions significantly contribute to the variation in chloroplast genome size in *Prasiolopsis* and *Watanabea* species [46,53]. Previous studies indicate that the intergenic regions of terrestrial algae chloroplast genomes tend to be larger than those of their aquatic counterparts [54]. A synteny analysis revealed numerous rearrangements and inversions in the intergenic regions (Figures S2 and S3). Therefore, the intergenic regions may play an essential role in the adaptive evolution of terrestrial algae.

### 4.2. Codon Usage Bias

Research on codon usage bias has been conducted in bacteria, plants, and animals [55]. This is the first study to systematically analyze the codon usage pattern of the *Cephaleuros* and *Trentepohlia* species. The advancement of next-generation sequencing technology has made it possible to gain a large number of algal chloroplast genomes, including those of Trentepohliales species, thus facilitating the study of codon usage bias. In the present study, we analyzed 10 chloroplast genomes of *Cephaleuros* and *Trentepohlia* genera to investigate codon usage patterns and the factors that influenced the codon usage bias.

In PR2 plot analysis, when only mutational pressure acts on the codons of chloroplast genes, all the points should be located in the center of the plot. It was obvious that our results showed an uneven distribution of genes in the four quadrants (Figure 4). The numerous data points deviate from the center, while a few points are situated near or on the center (Figure 4), indicating that codon usage patterns are jointly influenced by mutations and selection pressure, with natural selection playing a prominent role. Furthermore, in the vertical direction, the majority of genes were situated above the center line. In the horizontal direction, there were more genes on the left than on the right. Therefore, A was used more frequently compared to T, and C was used more frequently compared to G at the third position of codons.

In the ENC plot, a few genes were found to be close to the expected line, indicating that the codon bias of these genes was correlated to mutation pressure. However, the majority of genes were situated below the expected curve, revealing the actual ENc values were smaller than expected (Figure 5 and Figure 6). This indicates that codon bias was influenced by natural selection.

Neutrality analysis indicates that if there is a significant correlation between GC12 and GC3, and the slope of the regression line is near to one, it can be concluded that mutation pressure is the predominant factor influencing codon usage patterns. Conversely, if the relationship is not significant, then the slope of the regression line is close to 0, indicating that codon bias is primarily influenced by natural selection [25]. In the present study, there was no significant correlation between GC12 and GC3, and the slope of the regression line was close to 0 (Figure 7). Our results showed that the GC3 contents in the chloroplast genomes of *Cephaleuros* and *Trentepohlia* species were consistently less than 40% (Figure 7). This suggests a preference for codons ending with A and T. The PR2 plot and ENC plot revealed that natural selection plays a dominant role in determining the codon usage in the genera *Cephaleuros* and *Trentepohlia*, while mutation pressure plays a secondary role.

### 4.3. Adaptive Evolution and Positive Selection

Genome sequences of closely related species make it possible to quantify the frequency of positive and negative selection in the genome [28]. The Ka/Ks ratio serves as a measure of natural selection. The values of Ka/Ks < 1, =1, and >1 indicate negative purifying selection, neutral evolution, and positive selection, respectively [28]. In the genera *Cephaleuros* and *Trentepohlia*, the pairwise Ka/Ks ratios at the chloroplast genome level were significantly less than 1, indicating strong purifying selection (Figure 8A, Table S4). The average Ka/Ks ratio for the chloroplast protein genes analyzed in the 10 genomes was 0.0442. The majority of genes have Ka/Ks values between 0 and 0.01, indicating that most genes were subjected to purifying selection in order to maintain their conserved functions (Table S5). A small number of genes, such as *psbA*, *psbT*, and *rps9*, had Ka/Ks ratios around 0.5, implying the presence of potential positive selection sites.

The *Cephaleuros* species are parasitic or endoparasitic on the leaves or twigs of plants that inhabit shaded and humid environments [2]. We inferred that some genes in the plastids of *Cephaleuros* might have undergone adaptive evolution to adapt to shaded and humid environments. In our study, the branch-site analysis revealed positive selection in six genes (*atpF*, *chlN*, *psaA*, *psaB*, *psbA*, and *rbcL*) of *Cephaleuros* species (Figure 8C). These genes can be classified into three categories: photosystem subunit genes (*psbA*, *psaA*, *psaB*, and *rbcL*), ATP subunit genes (*atpF*), and chlorophyll biosynthesis gene (*chlN*).

Notably, four genes (*psbA*, *psaA*, *psaB*, and *rbcL*) associated with photosynthesis are under positive selection, and *psbA* encodes one of the two reaction center proteins in photosystem II. The reaction center of photosynthesis is photosystem II, which uses light energy to drive the oxidation of water, producing O_2_ and a proton gradient subsequently used to produce ATP [56]. The *psbA* is vital for the assembly or function of the photosystem II complex. The *psbA* gene was detected under positive selection in *Curcuma* and *Oedogonium* [57,58]. It is speculated to play an important role in the evolutionary adaptation of these organisms to different light intensities. Two photosystem I subunits (*psaA* and *psaB*) were under positive selection. The *psaA* and *psaB* bind P700 (the primary electron donor of photosystem I), as well as the electron acceptors A0, A1, and FX in photosystem I. Photosystem I is a plastocyanin/cytochrome c6-ferredoxin oxidoreductase, converting photonic excitation into a charge separation, which transfers an electron from the donor P700 chlorophyll pair to the spectroscopically characterized acceptors A0, A1, FX, FA and FB [59]. Notably, the *psaA* gene possessed the highest number of sites under positive selection, suggesting its pivotal role in the adaptive evolution of *Cephaleuros* species. The three positive selection sites in *rbcL* gene were discovered in *Cephaleuros* species. The *rbcL* gene encoded the large subunits of Rubisco, which plays an important modulating role in photosynthetic electron transport [60]. Previous studies revealed that the *rbcL* had also undergone adaptive evolution in Orchidaceae, Zingiberoideae, and Lardizabaloideae [61,62,63]. Genes related to photosynthesis are more likely to evolve adaptively in plants distributed in shady or sunny environments. In particular, *rbcL* and *atpF* are also under selection pressure in shade-tolerant *Oryza* species and shade-demanding *Panax* species, probably in response to low-light intensity [64,65]. *Cephaleuros* species are typically parasitic or endoparasitic on leaves or twigs of plants in wet and shady habitats. Therefore, the positive selection genes involved in photosynthesis may play a crucial role in the adaptive evolution of *Cephaleuros* to low-intensity light.

The *atpF* encodes the subunits of the H+-ATP synthase, which plays an essential role in ATP synthase and photophosphorylation during photosynthesis [66]. Positive selection on the *atpF* genes may contribute to meeting the energy demands of *Cephaleuros* during its adaptation to diverse environmental conditions. The *chlN* gene, together with *chlB* and *chlL*, encodes the light-independent protochlorophyllide oxidoreductase (LIPOR) proteins, which play a crucial role in the conversion of protochlorophyllide to chlorophyllide during chlorophyll biosynthesis in the dark [67]. The *chlN* genes were not universally distributed in plastids, and have been subjected to pseudogenization or lost in cryptophyte algae [68]. However, the gene remains in the *Cephaleuros* plastid, and is under positive selection. Therefore, it may confer an evolutionary advantage to the *Cephaleuros* species for survival and proliferation within its unique ecological niche.

### 4.4. Phylogenetic Analysis

The phylogenetic trees were constructed based on ITS rDNA and chloroplast protein-coding genes. In the ITS rDNA phylogenetic tree, *C. lagerheimii* formed a distinct lineage and was sister to other *Cephaleuros* species (Figure S4), which was consistent with the result based on the chloroplast protein-coding genes (Figure S5). The position of *C. diffusus* in the ITS phylogenetic tree was inconsistent with its position in the phylogenetic tree based on chloroplast protein-coding genes. *C. diffusus* was located in the *C. diffusus* lineage in Figure S4, while it was a sister group to *C. karstenii* and *C. virescens* in Figure S5. This may be due to the limited number of chloroplast genomes of *C. diffusus* in the database. Previous studies have confirmed that *C*. *virescens* was paraphyletic [9,69,70,71]. *C*. *virescens* was found to be monophyletic on the ITS rDNA phylogenetic tree, which could be attributed to our inadequate sampling. The non-monophyletic trait of *Cephaleuros virescens* was confirmed by the phylogenetic tree constructed using chloroplast protein-coding genes in this study. In Figure S5, *C. virescens* NC060531 was clustered with *C. parasiticus*, while *C. virescens* OQ848499 formed a sister relationship with *C. karstenii*. Previous studies speculated that the limited morphological characteristics, such as rhizoids and host invasion type, were the primary reasons for the incongruity between molecular phylogeny and morphological data in *Cephaleuros* [9,71]. Another possible reason is that the morphological and molecular evidence was not obtained from the same algal spot [71]. Therefore, sufficient morphological characteristics and correct sampling methods are crucial for delineating the boundaries of *Cephaleuros* species in future studies.

## Figures and Tables

**Table 1:** General characteristics and comparison of the chloroplast genomes of *Cephaleuros* and *Trentepohlia*. The three chloroplast genomes obtained in this study are shown in bold.

Species	GenBank Number	Size (bp)	Total Genes	GC (%)	tRNA	rRNA	CDS	IR
***C. lagerheimii***	OQ848500	480,613	94	29.5	27	3	64	lack
***C. diffusus***	OQ848501	383,846	94	33.4	27	3	64	lack
***C. virescens* FJ1315**	OQ848499	472,444	94	33.7	27	3	64	lack
*C. virescens* SAG 42.85	NC060531	314,936	95	36.1	28	3	64	lack
*C. karstenni*	NC060534	371,192	98	29.9	30	3	65	lack
*C. parasiticus*	NC060533	266,729	96	35.9	29	3	64	lack
*Trentepohlia* sp. BN17	NC060532	282,795	95	33.2	28	3	64	lack
*Trentepohlia* sp. YN1242	MZ334625	216,308	93	25.9	25	3	65	lack
*Trentepohlia* sp. YN1317	MZ334626	408,697	94	31.7	26	6	65	lack
*Trentepohlia odorata*	NC043776	399,372	97	29.8	30	3	63	have

## Data Availability

All newly chloroplast genome sequences in this study have been submitted to NCBI (https://www.ncbi.nlm.nih.gov/, accessed on 20 November 2023) with accession numbers from OQ848499 to OQ848501 listed in Table 1. The ITS sequence of *Cephaleuros diffusus* was also available in NCBI with accession number PP033476.

## Supplementary Materials

The following supporting information can be downloaded at: https://www.mdpi.com/article/10.3390/genes15070839/s1, Figure S1: Number of group I introns and group II introns. The top box is the number of group I introns, and the bottom box is the number of group II introns; Figure S2: Synteny comparison of *Cephaleuros* chloroplast genomes; Figure S3: Synteny comparison of *Trentepohlia* chloroplast genomes; Figure S4: BI tree of *Cephaleuros* species inferred from ITS rDNA sequences. The values of BI posterior probabilities (left) and MLbootstrap (right) are shown at the nodes. Support values of bootstrap/posterior probabilities >50/0.5 were shown. Scale bar indicates substitutions per site; Figure S5: ML phylogenetic tree based on chloroplast protein-coding genes. Maximum likelihood bootstrap values (1000 replicates) are given near the nodes. Scale bar indicates substitutions per site; Table S1: List of genes annotated in the three *Cephaleuros* chloroplast genomes. The number in brackets after the genes represents the copy number; Table S2: DCJ values of *Cephaleuros* chloroplast genomes; Table S3: DCJ values of chloroplast genomes of the genera *Cephaleuros* and *Trentepohlia*; Table S4: Summary of pairwise Ka/Ks ratios in the genera *Cephaleuros* and *Trentepohlia*; Table S5: The statistic of genetic Ka/Ks among the genera *Cephaleuros* and *Trentepohlia*; Table S6: The positive selection sites based on the branch-site model; Table S7: The partitions and best model for IQtree.
