# Supplementary material for: Comparative Analysis of Chloroplast Genomes in Cephaleuros and Its Related Genus (Trentepohlia): Insights into Adaptive Evolution
Source: Genes (Basel). 2024 Jun 26;15(7):839. doi: 10.3390/genes15070839 (PMC11275322; doi:10.3390/genes15070839)
Supplement: Supplementary file 1 [file genes-15-00839-s001.zip › supplementary materials/Caption of Supplementary Materials.docx]

**Figure S1:** Number of group I introns and group II introns. The top box is the number of group I introns, and the bottom box is the number of group II introns;

**Figure S2:** Synteny comparison of *Cephaleuros* chloroplast genomes;

**Figure S3:** Synteny comparison of *Trentepohlia* chloroplast genomes;

**Figure S4:** BI tree of *Cephaleuros* species inferred from ITS rDNA sequences. The values of BI posterior probabilities (left) and MLbootstrap (right) are shown at the nodes. Support values of bootstrap/posterior probabilities >50/0.5 were shown. Scale bar indicates substitutions per site;

**Figure S5:** ML phylogenetic tree based on chloroplast protein-coding genes. Maximum likelihood bootstrap values (1000 replicates) are given near the nodes. Scale bar indicates substitutions per site;

**Table S1:** List of genes annotated in the three *Cephaleuros* chloroplast genomes.

**Table S2:** DCJ values of chloroplast genomes of the *Cephaleuros* species;

**Table S3:** DCJ values of chloroplast genomes of the genera *Cephaleuros* and *Trentepohlia*;

**Table S4:** Summary of Pairwise Ka/Ks ratios in the genera *Cephaleuros* and *Trentepohlia*;

**Table S5:** The statistic of genetic Ka/Ks among the genera *Cephaleuros* and *Trentepohlia*;

**Table S6:** The positive selection sites based on the branch-site model;

**Table S7:** The potential positive selection test based on the branch-site model; Table S7: The partitions and best model for IQtree.
