# Supplementary material for: Comparative Analysis of Chloroplast Genomes in Cephaleuros and Its Related Genus (Trentepohlia): Insights into Adaptive Evolution
Source: Genes (Basel). 2024 Jun 26;15(7):839. doi: 10.3390/genes15070839 (PMC11275322; doi:10.3390/genes15070839)
Supplement: Supplementary file 1 [file genes-15-00839-s001.zip › supplementary materials/Table S1.docx]

| **Category** | **Group of genes** | **Name of genes** |
| --- | --- | --- |
| Self-replication | Large subunit of ribosomal proteins | *rpl2*, *rpl5*, *rpl12*, *rpl14*, *rpl16*, *rpl20*, *rpl23*, *rpl32* |
|  | Small subunit of ribosomal proteins | *rps2*, *rps3*, *rps4*, *rps7*, *rps8*, *rps9*, *rps11*, *rps12*, *rps14*, *rps18*, *rps19* |
|  | DNAdependent RNA polymerase | *rpoA*, *rpoB*, *rpoC1*, *rpoC2* |
|  | rRNA genes  tRNA genes | *rrl*, *rrf*, *rrs*  *trnA-TGC*, *trnC-GCA*, *trnD-GTC*, *trnE-TTC*(2), *trnF-GAA*, *trnG-TCC*, *trnI-AAT*, *trnK-TTT*, *trnH-GTG*, *trnL-TAA*, *trnL-TAG*, *trnM-CAT*(3), *trnN-GTT*, *trnP-TGG*, *trnQ-TTG*, *trnR-ACG*, *trnR-TCG*, *trnR-CCT*, *trnS-GCT*, *trnS-TGA*, *trnT-TGT*, *trnY-GTA*, *trnV-TAC*, *trnW-CCA* |
| Photosynthesis-related genes | Photosystem I | *psaA*, *psaB*, *psaC*, *psaI*, *psaJ* |
|  | Photosystem II | *psbA*, *psbB*, *psbC*, *psbD*, *psbE*, *psbF*, *psbH*, *psbI*, *psbJ*, *psbK*, *psbL*, *psbM*, *psbN*, *psbT*, *psbZ* |
|  | Chlorophyll biosynthesis | *chlI*, *chlL*, *chlN*, *chlB* |
|  | Cytochrome b6/f complex | *petA*, *petB*, *petD*, *petG*, *petL* |
|  | ATP synthase | *atpA*, *atpB*, *atpF*, *atpH*, *atpI* |
|  | Rubisco | *rbcL* |
| Other genes | Protease | *clpP* |
|  | Hypothetical protein | *ycf1*, *ycf3*, *ycf12* |
|  | Translation factors | *tufA*, *infA* |
|  | c-Type cytochrome synthesis gene | *ccsA* |

Table S1. List of genes annotated in the three *Cephaleuros* chloroplast genomes.
