# Supplementary material for: Comparative Analysis of Chloroplast Genomes in Cephaleuros and Its Related Genus (Trentepohlia): Insights into Adaptive Evolution
Source: Genes (Basel). 2024 Jun 26;15(7):839. doi: 10.3390/genes15070839 (PMC11275322; doi:10.3390/genes15070839)
Supplement: Supplementary file 1 [file genes-15-00839-s001.zip › supplementary materials/Table S2.docx]

Table S2. DCJ values of *Cephaleuros* chloroplast genomes.

|  | *C. virescens* SAG 42.85 | *C. virescens* FJ1315 | *C. karstenii* | *C. parasiticus* | *C. diffusus* | *C. lagerheimii* |
| --- | --- | --- | --- | --- | --- | --- |
| *C. virescens* SAG 42.85 | 0 | 41 | 40 | 10 | 35 | 31 |
| *C. virescens* FJ1315 |  | 0 | 29 | 39 | 27 | 25 |
| *C. karstenii* GD1942 |  |  | 0 | 39 | 21 | 22 |
| *C. parasiticus* GD1927 |  |  |  | 0 | 36 | 32 |
| *C. diffusus* HB1902 |  |  |  |  | 0 | 14 |
| *C. lagerheimii* GX1816 |  |  |  |  |  | 0 |
