# Supplementary material for: Comparative Analysis of Chloroplast Genomes in Cephaleuros and Its Related Genus (Trentepohlia): Insights into Adaptive Evolution
Source: Genes (Basel). 2024 Jun 26;15(7):839. doi: 10.3390/genes15070839 (PMC11275322; doi:10.3390/genes15070839)
Supplement: Supplementary file 1 [file genes-15-00839-s001.zip › supplementary materials/Table S3.docx]

Table S3. DCJ values of chloroplast genomes of the genera *Cephaleuros* and *Trentepohlia*.

|  | *C. virescens* SAG 42.85 | *C. virescens* FJ1315 | *C. karstenii* GD1942 | *C. parasiticus* GD1927 | *Trentepohlia* sp*.* BN17 | *C. diffusus*  HB1902 | *C. lagerheimii*  GX1816 | *Trentepohlia* sp.  YN1242 | *Trentepohlia* sp.  YN1317 | *Trentepohlia odorata* |
| --- | --- | --- | --- | --- | --- | --- | --- | --- | --- | --- |
| *C. virescens* SAG 42.85 | 0 | 41 | 40 | 10 | 38 | 35 | 31 | 39 | 39 | 68 |
| *C. virescens* FJ1315 |  | 0 | 29 | 39 | 28 | 27 | 25 | 25 | 29 | 66 |
| *C. karstenii* GD1942 |  |  | 0 | 39 | 31 | 21 | 22 | 31 | 33 | 64 |
| *C. parasiticus* GD1927 |  |  |  | 0 | 38 | 36 | 32 | 41 | 43 | 69 |
| *C. tumidae-setae* BN17 |  |  |  |  | 0 | 31 | 27 | 24 | 20 | 59 |
| *C. diffusus* HB1902 |  |  |  |  |  | 0 | 14 | 31 | 31 | 67 |
| *C. lagerheimii* GX1816 |  |  |  |  |  |  | 0 | 25 | 29 | 67 |
| *Trentepohlia* sp. YN1242 |  |  |  |  |  |  |  | 0 | 26 | 66 |
| *Trentepohlia* sp. YN1317 |  |  |  |  |  |  |  |  | 0 | 66 |
| *Trentepohlia odorata* |  |  |  |  |  |  |  |  |  | 0 |
