# Supplementary figures and images for: Comparative Analysis of Chloroplast Genomes in Cephaleuros and Its Related Genus (Trentepohlia): Insights into Adaptive Evolution
Source: Genes (Basel). 2024 Jun 26;15(7):839. doi: 10.3390/genes15070839 (PMC11275322; doi:10.3390/genes15070839)

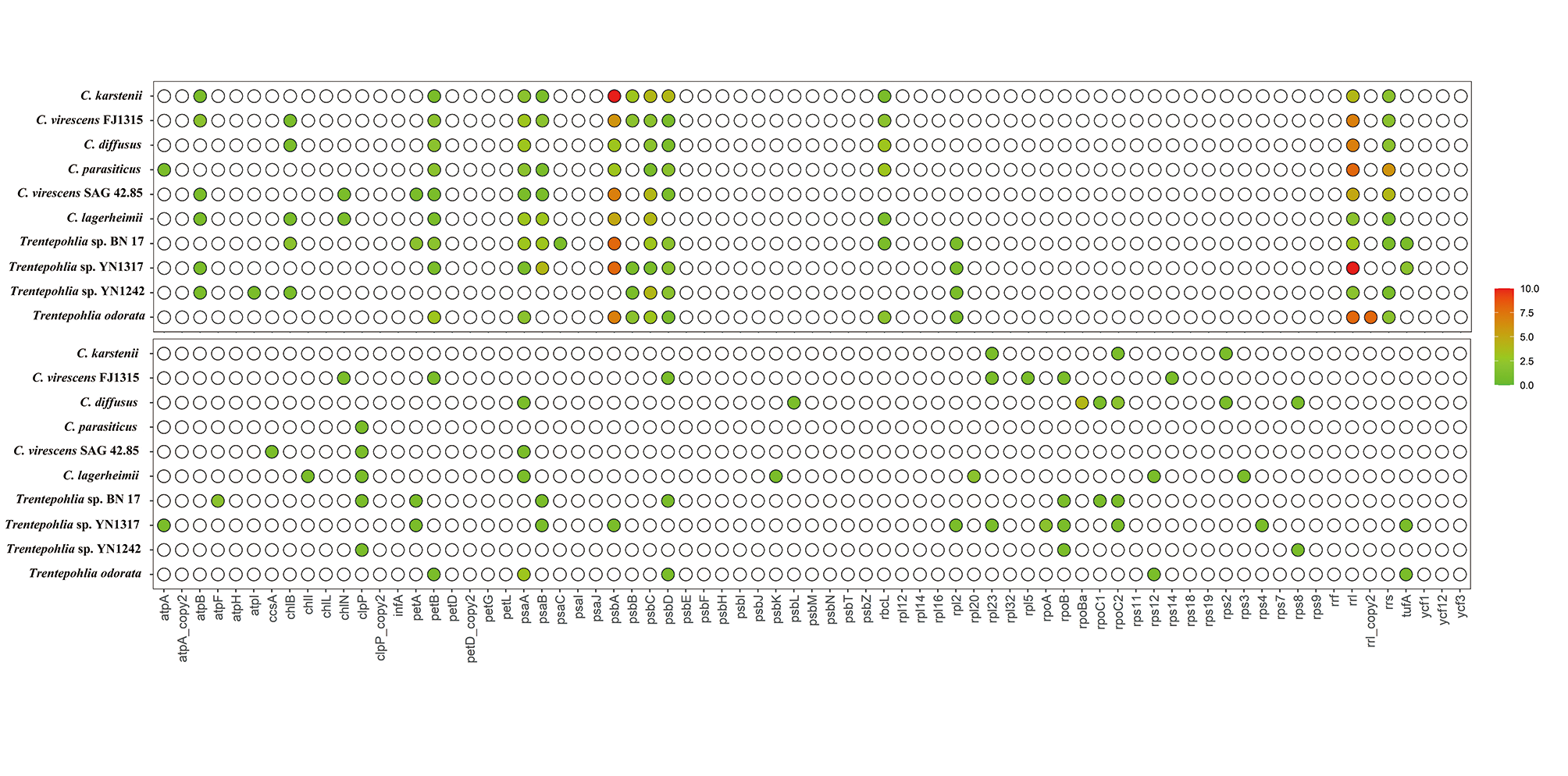

Supplement: Supplementary file 1 [file genes-15-00839-s001.zip › supplementary materials/Figure S1.tif]

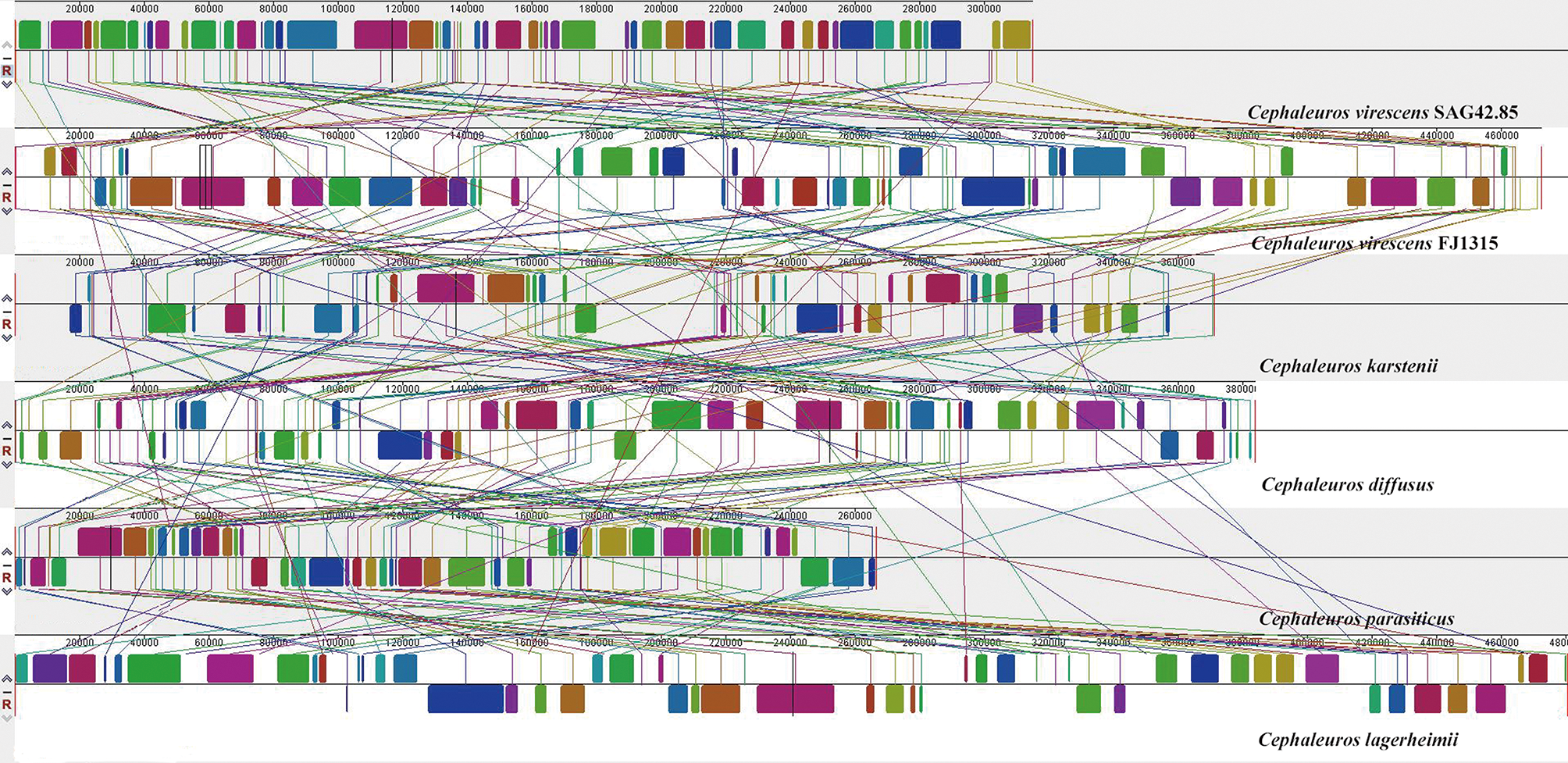

Supplement: Supplementary file 1 [file genes-15-00839-s001.zip › supplementary materials/Figure S2.tif]

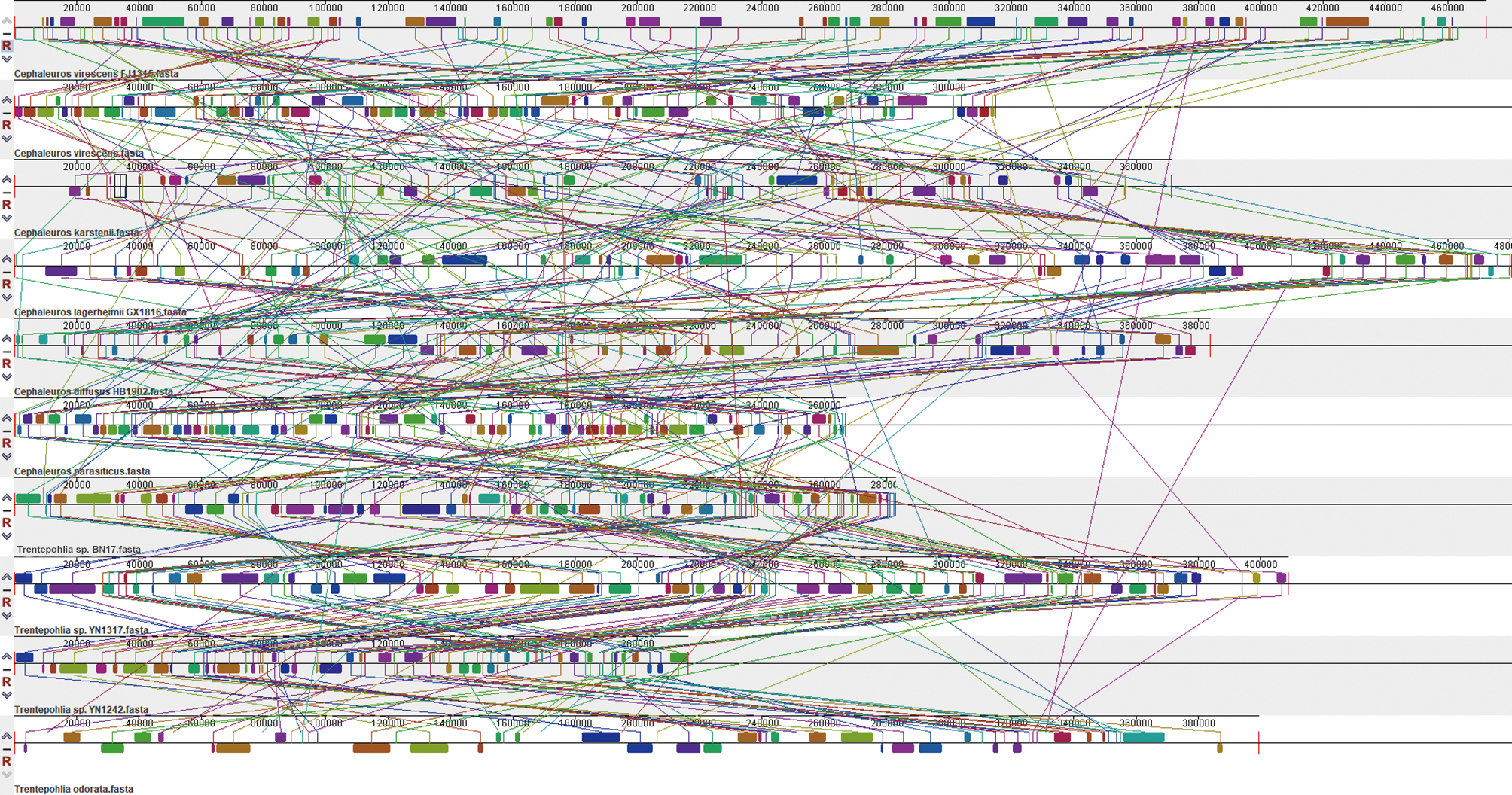

Supplement: Supplementary file 1 [file genes-15-00839-s001.zip › supplementary materials/Figure S3.tif]

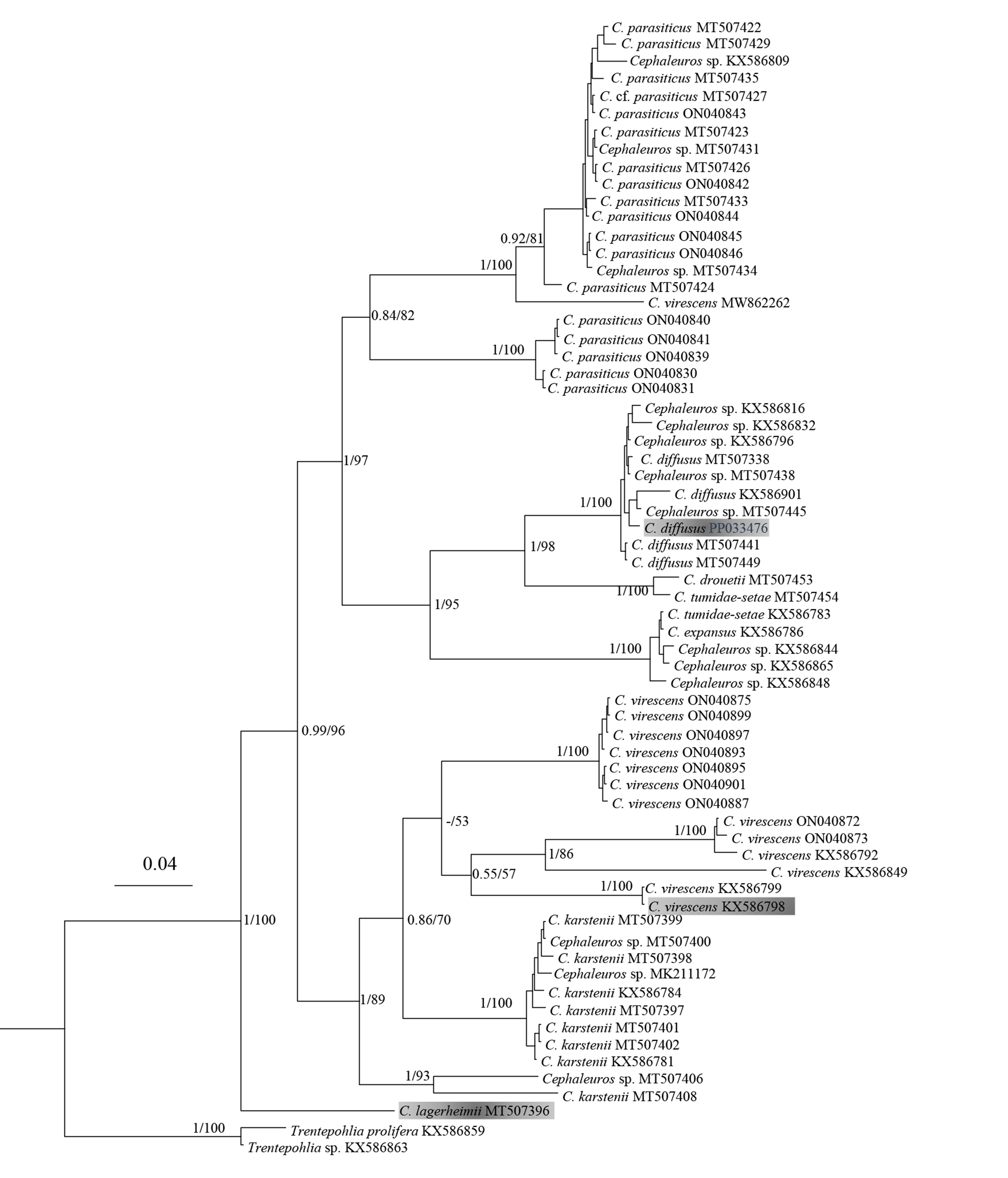

Supplement: Supplementary file 1 [file genes-15-00839-s001.zip › supplementary materials/Figure S4.tif]

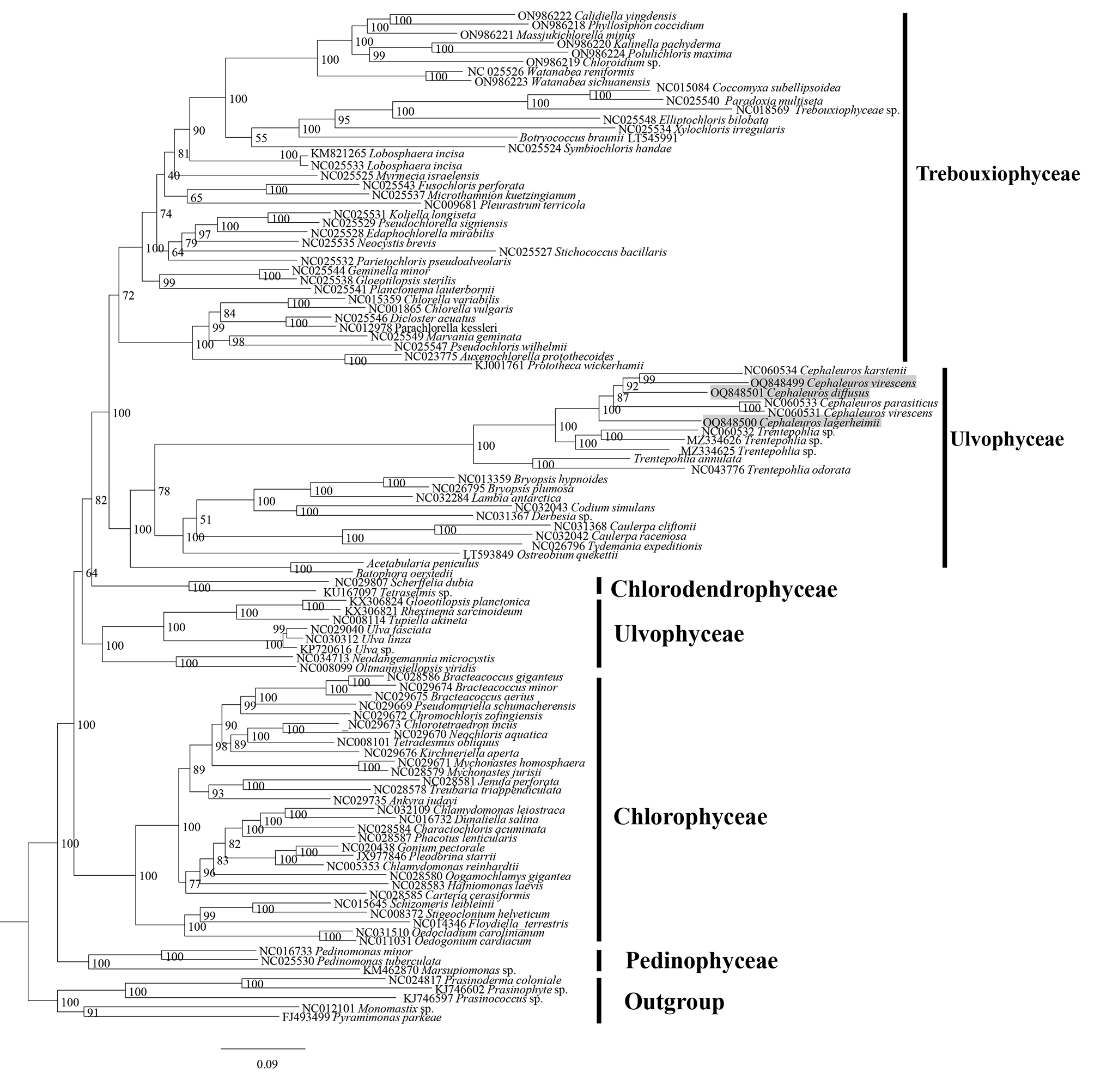

Supplement: Supplementary file 1 [file genes-15-00839-s001.zip › supplementary materials/Figure S5.tif]
